# Pennsylvania State Veterinary Medical Association

**Published:** 1898-04

**Authors:** 


### SOCIETY PROCEEDINGS.

### ANNUAL MEETING

OF THE

### PENNSYLVANIA STATE VETERINARY MEDICAL ASSOCIATION.

AS TOLD BY A STAFF CORRESPONDENT.

BEAUTIFUL weather and bright environments made auspicious the opening of the Keystone State organization's sixteenth annual meeting, at the Veterinary Department of the University of Pennsylvania, at 10 o'clock on the morning of March 8, 1898.

President Rayner looked upon a larger number of faces than had ever been gathered at a single meeting in Pennsylvania's history in the veterinary profession. The roll-calls on the 8th and 9th showed the presence of more than sixty members, as follows: Drs. John W. Adams, Francis Allen, Philadelphia; J. C. Bartholomew, Berwyn; W. G. Benner, Doylestown; Henry Bowers, Philadelphia; John L. Bradley, Mercersburg; Francis Bridge, Philadelphia; J. F. Butterfield, South Montrose; A. O. Cawley, Milton; M. J. Collins, Myerstown; M. E. Conard, West Grove; H. B. Felton, Olney; J. T. Ferley, Philadelphia; J. C. Foelker, Allentown; Robert Formad, Philadelphia; Robert Gladfelter, Philadelphia; Charles T. Goentner, Bryn Mawr; S. J. J. Harger, Philadelphia; Walter L. Hart, Philadelphia; F. F. Hoffman, Brookville; E. Hogg, Kirkwood; W. Horace Hoskins, Philadelphia; Joseph D. Houldsworth, Manayunk, Philadelphia; Horace P. Keely, Schwenksville; Z. S. Keil, Perkasie; Edward L. Kellner, Philadelphia; W. H. Knight, Kennett Square; W. S. Kooker, Philadelphia; J. M. Kuhn, Mercersburg; S. D. Larzelere, Jenkintown; Charles Lintz, Chester; L. O. Lusson, Ardmore; C. J. Marshall, Philadelphia; J. C. Michener, Colmar; W. B. E. Miller, Passaic, N. J.; E. S. Moyer, Blooming Glen; James T. McAnulty, Philadelphia; J. C. McNeil, Pittsburg; Otto G. Noack, Reading; J. H. Oyler, Harrisburg; Leonard Pearson, Philadelphia; William P. Phipps, Lionville; A. W. Radley, Bethlehem; G. B. Rayner, Manayunk, Philadelphia; Thomas B. Rayner, Chestnut Hill, Philadelphia; James B. Rayner, West Chester; John B. Raynor, Milestown, Philadelphia; N. E. Reinhart, Pottstown; W. L. Rhoads, Lansdowne; W. H. Ridge, Trevose; James T. Ross, Frankford, Philadelphia; J. W. Sallade, Pottsville; Charles Schauffler, A. F. Schrieber, B. F. Senseman, Philadelphia; H. W. Turner, Lahaska; E. E. Terry, Holmesburg; B. M. Underhill, Media; R. G. Webster, Salem, N. J.; Charles Williams, Philadelphia.

Ten new members were added to the roll, of whom the following were present at the meeting: Raymond Barber, V.M.D., Newtown; H. D. Hackler, V.S., A. N. Lushington, V.M.D., E. M. Ranck, V.M.D., and W. G. Shaw, V.M.D., Philadelphia; J. Stewart Lacock, V.M.D., Allegheny; and George W. Swank, V.M.D., East Mauch Chunk.

The visitors from all over the State, from New York, New Jersey, and

Delaware were greater in number than ever before, and among them were to be noted: E. B. Ackerman, D.V.S., Brooklyn, N. Y.; H. P. Eves, V.M.D., Wilmington, Del.; John Faust, V.S., Poughkeepsie, N. Y.; William Herbert Lowe, D.V.S., Paterson, N. J.; C. E. Magill, V.M.D., Haddonfield, N. J.; Mr. Joseph Matlack, Maple Shade, N. J.; James M. Mecray, V.M.D., Maple Shade, N. J.; J. Cheston Morris, M.D., M. P. Ravenel, M.D., and Dr. Woodward, of City Board of Health, Philadelphia, Pa.; Drs. U. S. G. Bieber, Kutztown; J. M. Eshleman, Parkersburg; J. Z. Hillegass, Red Hill; N. C. Lazarus, Stroudsburg; Charles Leonard, Dover; A. R. May, Boiling Spring; L. E. Mead, Tunkhannock; W. A. Meredith, Corry; Harry B. Rayner, Norristown; E. E. Tower, Montrose; Charles J. Waidner, Hellertown, Pa.; Mr. George Abbott, Drs. J. F. Connor, M. W. Drake, Alexander Glass, Mr. G. H. Gordon, Drs. James Johnston, A. L. Lehman, C. H. Magill, H. D. Martein, Frank C. McCurdy, W. B. Montgomery, E. Stanton Muir, Edward O'Connor, John A. Pearson, J. Price Vasey, Otto von Lang, Mr. J. J. Teufel, Mr. F. R. Trowbridge, Philadelphia; Mr. S. G. Brosius and W. H. Smith, Jr.

The attractions of so many prominent practitioners, to hear them discuss the excellent papers, reports, etc., proved a strong incentive to the student body of the University's Veterinary Department, and noted as being present were: Messrs. L. L. Cheney, G. E. Chesley, E. L. Cornman, A. E. Cunningham, M. Jacobs, Joseph Johnson, Jr., Philip K. Jones, L. D. Horner, H. Hoopes, William Hughes, C. F. Keiter, Bassett Kirby, C. G. Land, S. W. McClure, John H. McNeall, Joseph N. Megary, Jr., J. P. Miller, L. A. Nolan, W. E. Phillips, John J. Repp, J. E. Spindler.

After the reading and adoption of the minutes of the semi-annual meeting, President James B. Rayner, of West Chester, one of the earliest practitioners of Pennsylvania, delivered his annual address. It was listened to with marked attention, and was worthy of the occasion.

*The President's Address.*

The assembling to-day of our Association in annual convention is an occasion worthy of more than passing notice. It marks another period of our history, and it is well for us to look back and note how well the year's work has been done. I am proud to say that I have had the earnest support of my fellow-officers, and that much work has been done looking to the welfare of our calling. Our semi-annual meeting at Franklin was, perhaps, the most successful we have held during the past ten years, in attendance, in scope of work, in pleasure, in increased membership, and everything that adds to our progress.

When I recall the early history of the profession in Pennsylvania, at a time when many of you were not born, and that the Philadelphia Veterinary College was the first lawfully chartered veterinary college in America, and note the same intense interest in professional advancement, I am more than proud of our record in the profession. While this college remained but a few years in active teaching, still its influence was good; it led to the first movement to establish a veterinary chair in the University of Pennsylvania, and two of the foremost pioneers in medicine and surgery urged its importance and advocated its establishment. I refer to the honored names of Professors Gross and Elwyn, who joined with Professors McClure and Jennings of our own branch of medicine. At that time our University was on Ninth Street, and the little room it had, made it next to impossible to establish this department. The Philadelphia College, with but few students each year, not enough to support it, without State or private assistance, was compelled to close during the session of 1865-1866. The University, moving to West Philadelphia a few years later, still appreciating the importance of this branch of scientific study, kept fully alive to its necessity, and established this department in 1883, with our esteemed colleague, Prof. R. S. Huidekoper, as Dean, with Drs. Zuill, Hoskins, and Glass as instructors, and its work of recent years in sending forth well-equipped and efficient veterinarians is known to you all.

In association work our State has an enviable reputation. It had a number of its practitioners among the roll of organizers of the United States Veterinary Medical Association, formed in 1863. The aim, then, of contributing to the diffusion of true science, and particularly the knowledge of veterinary medicine and surgery, is still its motto and field of work, and to-day with its membership of over four hundred, are to be found working along, shoulder to shoulder, many of the earnest workers in veterinary science in the Keystone State.

The first Pennsylvania Association was organized in 1865, and it continued in more or less active existence until 1883, when its most active members joined with others in the formation of this Association, which chose for its first President our still active and worthy member, Dr. James W. Sallade. With many graduates of the American, New York, and Canada Colleges, and a large number of excellent practitioners, this organization has marched on for almost a score of years, more earnest and faithful to all its duties with each added year of its existence, until it has a well-earned reputation, second to none, for good work.

The Keystone Veterinary Medical Association of this city and vicinity, organized October 7, 1882, has continued in active existence ever since its first meeting, and added each year its assistance to the welfare of the veterinary profession. More active than ever, its work during the present and past year has made each of its members justly proud of its labors and achievements.

With our other local associations of the Schuylkill and Wyoming Valleys, most of whose members are active in this organization, I assure you, fellow-members, I am justly proud of the record made by Pennsylvanians, and I am more than proud of the honor of your recognition as President of such a body of earnest men, and after an active career of nearly forty years in the pursuit of veterinary practice, the greatest honor I could wish for has been bestowed upon me by this organization, and at a time when illness prevented my presence, but your recognition and esteem was never worn more proudly and with more earnest and devout wishes for the good of you all than my whole heart has wished for the advancement and welfare of one and all of you.

I now welcome you all to Philadelphia, and invite your close attention to the excellent programme that has been prepared for you, and to every enjoyment of it possible.

The nomination of officers for the ensuing year was a feature of much interest, and for the first time in the Association's history the sentiment seemed to prevail that the office of President should go to the western end of the State, and while the warmest appreciation was exhibited on every side for one of the most popular members of the eastern section of the State, whose name was presented, thus placing in the field Dr. George B. Jobson, of Franklin, and Dr. M. E. Conard, of West Grove, the latter before the vote was cast made one of the most grateful concessions by the withdrawal of his name, and thus made unanimous the selection of the Mayor of Franklin for President. It was a happy solution for all those who fully appreciated the worth of Dr. Conard, but who felt the time had come to yield to the prevailing sentiment that the chief office should rest this year in the west. The selection of officers resulted in the following choice: President, Dr. George B. Jobson, Franklin, Pa.; for First Vice-President,

Dr. J. Curtis Michener, of Colmar; for Second Vice-President, Dr. J. F. Butterfield, of Montrose; and for Third Vice-President, Dr. F. F. Hoffman, of Brookville; for Recording Secretary, Corresponding Secretary, and Treasurer, the outgoing officers, Drs. Jacob Helmer, W. L. Rhoads, and Francis Bridge, were recalled to the duties of these offices, which had been so well filled during the past year, and a unanimous vote was accorded them. By virtue of unforeseen circumstances at the last moment, the Recording Secretary, Dr. Jacob Helmer, was detained at home, and in accordance with the rules, this work fell to the lot of the Corresponding Secretary, who found his hands full at all times during the very busy meeting.

Some ten names were presented for places upon the Board of Trustees, resulting in the election of the outgoing President, Dr. James B. Rayner —a plan to be followed that the Association's interests may have for another year the value and aid of the retiring chief officer—and for the other four, Drs. W. Horace Hoskins, of Philadelphia; W. H. Ridge, of Trevose; J. C. McNeil, of Pittsburg, and James W. Sallade, of Pottsville, proved to have the highest number of votes, thus placing the Association interests well geographically and among those who have for many years been ardent in Association work and professional advancement.

The Board of Trustees, with Drs. W. H. Ridge, W. Horace Hoskins, and S. J. J. Harger present, were reinforced by appointment of the President, with the assistance of Drs. Thos. B. Rayner and Robert Gladfelter, and after a half hour's recess, during which time the Secretary and Treasurer were active in collecting dues, reported with a favorable recommendation the names of the following applicants: Drs. Wm. G. Shaw, A. N. Lushington, Edward M. Ranck, of Philadelphia; J. Stewart Lacock, of Allegheny; G. W. Swank, of East Mauch Chunk, graduates of the University of Pennsylvania Veterinary Department. Drs. H. D. Hackler, of Philadelphia; M. W. Keck, of Slatington; S. B. Bishop, of Carlisle, graduates of the Ontario Veterinary College. Dr. R. J. Mumma, of Hanover, graduate Veterinary Department, Columbia University, and Dr. R. G. Rice, of Towanda, graduate New York College of Veterinary Surgeons. All the recommendations of the Board were approved and the above applicants elected to membership in the Association, those present being introduced by the President.

Corresponding Secretary Rhoads made a brief report, accompanied with many letters of regret from members and others, also the resignation of Dr. P. K. Driebelbis, which was referred to the Board of Trustees, who finding nothing against the member, his dues paid, recommended its acceptance, which was adopted.

Treasurer Bridge made a partial report, which was supplemented by the committee directed to audit the late Treasurer's account reporting his books in proper order, the bills directed to be paid, properly indorsed with accompanying orders, and a balance due the Association of $68.27, all of which was approved pending final audit and settlement.

Committee reports proved of unusual interest. Some three of the Committee on Intelligence and Education offering specially well-prepared reports, which are as follows:

*Report of Committee on Intelligence and Education.*

When we look back and see what changes have been wrought in our profession we wonder how they were brought about. The demand to-day is for more and better education. In all fields of activity competition has become so great that the uneducated find it difficult to gain a livelihood. We do not intend to review the past, but we cannot refrain from commenting on the great difference in the demands of our colleges to-day in regard to entrance-examination. Can the examination be too high? We think so. We, to-day, have a much better grade of men entering our colleges than ever in the past; our colleges also are advancing very rapidly, adding more and better teachers to their staff. While these colleges are not self-supporting, generous public donations enable those who desire to gain an education. If it were not for this general interest of the public, with that kindly feeling toward us, we would advance in a much slower manner.

As we have only one veterinary school in this State, we are pleased to note advances made by it. Dr. Leonard Pearson has been made Dean. This is as it should be. While the department has been managed by an able man, and a good friend to the veterinarian, we cannot but think that the department will advance, and the students take a greater interest in it by having such an able veterinarian at its head. Dr. W. Horace Hoskins has been added to its staff of teachers, lecturing on jurisprudence, ethics, and business methods; this has added materially to the strength of the course. To-day the study of bacteriology is all important, and the needs of a good teacher. This school is fortunate in securing the services of Dr. M. P. Ravenel as teacher. Our member, Dr. C. J. Marshall, has the appointment of demonstrator of theory and practice of medicine. This school is working very harmoniously, and there exists the kindest feeling toward it by the public.

Other schools are doing their good work, advancing in a rapid manner. Some of our New York schools feel that they are handicapped by the law in that State governing the entrance-examination. We fear this will debar many men who would in later life prove to be able practitioners. Education takes in much more than answering a few questions that the examiners happen to ask, or what in their judgment one should know. Also some minds are capable of being taught in special branches much easier than in others, thus one person may become an expert surgeon, yet never could have become a classical scholar. We would not debar anyone from taking a course in veterinary medicine on account of his previous education, but only advise him of his chances, then if he takes the risk and passes his final examination satisfactorily, he should receive his diploma.

Our journals are one of the greatest sources of education that the veterinarian could be interested in. While our days at college are over to the majority of us, we still need instruction. Man can never remain in one place, nor can he remain at one degree of intelligence; he either progresses or goes backward. None want to be relegated to the past because of not keeping abreast of the times, so we depend upon our journals to point out our deficiencies. Of course, all have noted their great advance this last year or two. They are better edited, better printed, and have better articles than ever in the past. There is one point we see extended, that is, personal items and news. We want to know what our friends are doing and what is transpiring in our veterinary world. We hope that none of our members attempt to practice without our journals; they are as essential as medicine. We would like to see a few pages added monthly, devoted to judging of livestock. If there is one branch the veterinarian is deficient in more than another it is the judging of pedigrees and points of excellence of breeds. This should not be so. The judges of our fairs, etc., should be taken from the ranks of the veterinarian when such qualified men can be procured. Our students should be taught to judge by points of excellence, and give their reasons for it. To quote from an article written by Dr. Curtis, who says, "The judging at some of our own State fairs, where we expect a higher grade than at a county fair, is often simply a burlesque. At a State fair recently held, one of the judges, who assumed a degree of arrogance and importance equal to several ordinary men, and wisdom superior to several ordinary owls, did not know anything about swine-herd books, or standards, or requirements of associations to constitute thoroughbreds, but he ' knew a good hog and one which suited him.' Under the dictation and awards of such a judge the exhibitors of all breeds stood back in disgust, and let the thing run. How could anyone learn from such a decision in regard to characteristics, or qualities of any breed, or the valuable and best features in breeding. I would make the exhibit of as great practical value as possible, as this would add to the attractions of the fair; when people found out that teaching by object-lessons would take place, and by noted and accepted teachers, they would flock around the rings where stock was being judged, and the pens, to compare the points and evidence of value." Now, we often hear our veterinarians say, "the veterinarian is the proper person to judge stock." We ask, are they qualified? Do our schools teach them so as to be efficient judges? The veterinarian of the future will have to be educated on more branches, and as his services are called by men high in the art of breeding, his abilty is often judged by his conversation in regard to breeding or points of excellence; the mere individual ideas of perfection are oftentimes worse than no ideas. Our journals could teach us a vast amount on this subject. When a subject appears before us often that we know very little about, it shows us our weakness, and stimulates us to action; even the publishing of the questions of an examination oftentimes fills one with fear that he is regressing. How often do our journals notify us that we have forgotten something or that we are not as qualified as the times demand, and make us devote our spare hours to study.

New books are second only to our periodicals. During the last year we have had additions that are worthy of high commendation. Dr. Alex. Glass, a member of this Association, has added a translation of Professor Müller's *Diseases of the Dog*. Dr. John Adams, also one of our members, has added a translation of A. Lungwitz's text-book on *Horseshoeing*. Dr. W. E. A. Wyman is the author of *Clinical Diagnosis of Lameness in the Horse*. Dr. George G. Van Mater has contributed *Veterinary Ophthalmology*. The *Veterinary Blue-Book*, by Dr. R. S. Huidekoper; *Diseases of Swine*, by D. McIntosh; *Exercises in Equine Surgery*, P. J. Cadiot, translated by A. W. Bitting; *Biological Products Indicated in Veterinary Practice*, by H. K. Mulford Co.; *Reports of Bovine Tuberculosis*, by M. H. Reynolds; *Handy Manual of Veterinary Medicine*, by M. Stalker; *A Compend of Veterinary Obstetrics*, by W. H. Dalrymple; the third edition of Walley's *Guide to Meat-inspection; Text-Book of Comparative Pathology and Therapeutics of Man and the Domestic Animals*, for the veterinarian, physician, and student, in four volumes, by George Schneidenmuhl, in German.

The people of this country are realizing that we are advancing. We believe nothing has educated the public in this direction faster than our Association. We see in our daily papers, farm journals, etc., that our profession is being honored in many ways. In the *Rural New Yorker*, of a late issue, the following was printed: "The New York State Board of Health has been expending a little money in testing the dairy herds at State institutions. This serves to protect the inmates from diseased milk, and sets a good example. The article on our first page tells how these tests are conducted. While we are thinking about the State Board of Health, it occurs to us that a veterinarian or two would not be out of place upon it. Very likely, it must have to pass upon the fitness of men for herd-testing, and physicians do not get such training in comparative medicine as would be useful in that event. Then our brothers of the yoke and stall have many of our ailments, and we theirs." This shows us that the farmer does not dread the appearance of the veterinarian, as some prophesied a few years ago, when the examination of tuberculous animals was commenced, but are looking to us to help the public, and are placing the confidence in us that is justly due our profession. Our associations are educators that many fail to make use of.

Few realize the importance of being members of our associations. We see it in this State. It has always been a wonder to me why the majority of our profession remained outside the ranks of membership, when the interests of one was the interest of all. In some occupations the interests are different in different sections. What suits one neighborhood does not suit another. Organizations conflict with one another, while with us we have the same questions for all points. We deem it the duty of every qualified veterinarian to belong to our National Association, so as to give it power and recognition. We must not look to self-interest entirely. It may not benefit us directly. If a small proportion of the profession has advanced the standing so fast in these last few years, how much faster and easier could they have done it with your help? United, we progress and are recognized. Divided, we become "hoss-doctors." W. H. R.

Our men should be more active in national legislation that especially refers to our profession; such laws as involve the employment of our men in the meat-inspection and quarantine service and experimental work. Every veterinarian should, through his Congressman, keep in touch with the work of the Bureau of Animal Industry, by having his name placed on the list of those to receive the bulletins and reports of the Bureau. We note the advancing intelligence of the public on sanitary questions, milk- and meat-inspection, and the duty of the veterinarian in spreading this education. Our duties in aiding the State Board of Health measures, and thus increasing their interest in similar lines, especially interest us. Gov. Hastings deserves credit for the appointment and recognition of Dr. Benjamin Lee as health officer. Our interest in the milk question involves the commercial aspect in so far as proper methods for its safe distribution, but more especially in its production from healthy animals and under proper sanitary conditions. Our veterinary milk-inspectors should be more active in advancing legislation along such lines as have been long indicated as destined to remedy many of the grave existing defects which the profession looks to them to direct and secure, that this work may be extended and reach a higher state of perfection, whereby the field of work of our profession may be enhanced and enlarged. Progress in this direction has been too slow, indeed. Our profession has been more willing to follow than eager to lead. The excellent work of our State Sanitary Board has been aggressive, yet conservative, in its methods, and much of its work has received marked attention and recognition in many other States. Its bulletins have been carefully and thoughtfully prepared, and it has been a source of gratification to note their reception by our own people as well as by the authorities in other States. We are justly proud of the work done by this Board, and rejoice in what it has accomplished, and while Pennsylvania is more than willing to extend a helping hand to sister States, we are extremely pained to note that those charged with the same line of work in the Empire State should be so forgetful of its duty, as well as its obligations to our Board, by pirating from the tuberculosis bulletin issued by our Board, clothing it with evidence that would lead one to believe it emanated from the authorities of that State, no credit being given to the officers of our Board under whose direction it was first produced.

In the way of new books we have a number that have emerged from the printers' hands during the past year. Among others, the translation from the German of Müller's *Diseases of the Dog*, by Dr. Alex. Glass, the first scientific work on canine pathology that has been presented to the English-speaking and reading veterinarian. A *Clinical Diagnosis of Lameness*, prepared by W. E. A. Wyman, covers a very interesting and important part of our work, and its presentation in this form will be a very great aid to the everyday practitioner. Kobert's *Toxicology*, translated by Prof. Friedburg, of New York City, brings to us one of the most excellent works in the German language on this all-important subject. The arrangement is well adapted for quick consultation and prompt decision as to the proper course of treatment. The *Veterinary Blue-Book* has just reached us, and is thoroughly characteristic of its compiler, our colleague and late President, Prof. R. S. Huidekoper. It embodies in every way much more than it promised—the incorporation there of the various laws, national and State rules, regulations, and officers of so many important organizations all over the country in which we are much interested; the regulations of all common carriers, railroad, express, steamship, etc., make it an invaluable ready-reference book; the long list of names of the veterinary profession in New York State, and the more prominent ones throughout the United States, with much information about them; the officers of the National and State organizations, and much other matter of equal importance and interest. One's office desk will be incomplete without it. Another work that has come to us on an all-important subject, that of *Veterinary Ophthalmology*, by George G. Van Mater, deals with a subject of the utmost importance in equine and canine pathology, and it is unfortunate that the author has clung so closely to human ophthalmology, and, with a limited experience in comparative practice, has not given the prominence to the consideration of those diseases of so much daily practical importance to us in our profession. It is to be hoped that in the revision of this book these grave defects may be remedied, or that it may suggest the need of a book fully covering the subject from a wholly veterinary standpoint. A shorter and more concise work on veterinary obstetrics, especially adapted for students and their instruction, has been brought forth under the direction of Dr. W. H. Dalrymple, who as an instructor realized the need of such a work. The well-known ability of the author as an able practitioner, as well as instructor in several of our agricultural colleges, peculiarly fitted him for the preparation of such a work, and we are sure it will be equally well appreciated by those it is destined to benefit. We are well pleased to note the improved condition of our veterinary journals, which should be more generally supported.

W. S. K.

*Report of Dr Otto G. Noack, of the Committee on Intelligence and Education.*

The veterinary profession has difficult and important duties to fulfil—saving the wealth of the nation and protecting the lives of the people against diseases transferable from animal to man, therefore demanding the highest standard of education, preliminary and collegiate, from the students who intend to join the ranks of this high and noble profession. Experience has shown only men whose minds were well trained, and who had gained a thorough education, were able to promote the profession, and I am glad and proud to say that our State has quite a number of such men, men who with higher education have the welfare of their profession at heart, they being the prime movers of a higher standard in veterinary science, not only in our Commonwealth but in the whole United States.

I am sorry to state that one point is neglected. The preliminary education which is asked for matriculation in the Veterinary Department of the University is not high enough, and it is time to follow the example of the Empire State, where a thorough preliminary education for entering the veterinary college is required. Such a move would place us on the same basis with the European colleges and make the profession in this State equal to the standard of any other profession. At least a proper knowledge of Latin and Greek should be demanded before entering college, so as to understand the new and strange expressions the student hears in the lectures. If this had been asked many years ago we would not have such a conglomeration of technical terms as we have at present, where, for instance, apoplexy, paralysis, and paresis, etc., are thrown together, each word having a different meaning. Just think, parturient apoplexy, where paresis should be used, or whenever you hear this man had a slight stroke of apoplexy, where paralysis is in its place. Don't it make you laugh to hear such a mixture of expressions? Or what do you think of a man using paralysis for azoturia? This really shows the man that mixes such expressions has hardly any knowledge of veterinary science, and if he has, it is very poor. Therefore, we have to make efforts to raise the standard of matriculation into the college. Only by this can we expect to promote our profession socially and intellectually, when admitting only thoroughly educated men to enter the field of veterinary practice.

The Board of Examiners will do good work if not only inquiring of the scientific equipment of applicants of veterinary science, but also in their general education.

*Report of Dr. H. B. Felton, of Committee on Intelligence and Education.*

In reviewing the condition of our veterinary schools during the last year we find they have been sympathetically affected by the depreciation in the price of horses and by the widespread use of the trolley and bicycle; these factors, by rendering the outlook of the veterinarian less promising, have deterred many from matriculating, and as a consequence the entering classes have been greatly reduced in size. The lengthening of the course and the increased requirements in some of our schools have also had a deterring effect, so that we see no immediate danger of the profession becoming overcrowded. We note as a distinct advance the recent introduction of ambulatory clinics in the Veterinary Department of the University of Pennsylvania. These clinics are conducted by Dr. Hoskins, who takes the members of the senior class to see patients in his regular outdoor practice, where the appliances for treatment and the surroundings are radically different from what they are in a well-equipped veterinary hospital. The experience gained is extremely valuable, being that which a young practitioner usually gains through much tribulation in his first and second years of practice.

Great opposition has been raised in New York State by the action of the State Examining Board in making the standard of entrance so high. We have been informed that no less than eight bills are before the Legislature at the present time for the purpose of having the standard lowered.

We deprecate the establishment of the Grand Rapids Veterinary College as being unnecessary and a detriment to our profession. Being a two-year school, with a term of five months each, it is not possible to turn out men properly equipped for the requirements of our profession, which are constantly becoming more exacting. Beside this, it is in too close proximity to the Toronto school, which is a two-year school of very much higher standing.

We call the attention of our Association to reports of cases from time to time in some of our agricultural and stock journals, as they tend to make our profession ridiculous. One case recently reported was that of a mule which exhibited very peculiar brain-symptoms that greatly puzzled the attending doctor (?), and upon the death of the animal an autopsy was made, and the cause of death found to be a live mouse, which had run up the nostril and penetrated into the brain! We might well ask, which head was the more empty, the mule's or the man's? In another case, that of a horse, the mysterious cause of death was found to be gall-stones pressing on one of the main arteries. We might look on this as a case of transference of the brain to gall, but not on the part of the horse.

The work of the State Livestock Sanitary Board may also be considered as coming within the limits of the field of this committee. It has to do in many ways with intelligence and education. It is generally recognized that any attempts to control diseases of man or animals are almost hopeless when there is not widespread sympathy with the work. Sanitarians always strive for popular support. Efforts to enlist the co-operation of the public are not based on narrow grounds of petty policy, but are desired because they insure the permanency and efficiency of the work. Where active antagonism exists in relation to any sanitary measures, both the work and the agents are placed at a sad disadvantage. It is like fighting a battle in the country of the enemy. The first thing, therefore, for the State Livestock Sanitary Board to do was to enlist the support and assistance of the livestock owners. It could not do this by extremely radical measures necessitating a wide departure from established systems of management and business. Its duty was to point out the dangers that existed, the sources of disease and loss, and offer its services to the sufferers therefrom. So much misrepresentation in reference to the veterinary profession and to all veterinary measures has occurred; some sensational "yellow" agricultural papers have fostered and exaggerated these statements and impressions so much that when the State Livestock Sanitary Board was established a single false policy would have thrown the whole work into disrepute and have done incalculable harm to the subject of veterinary sanitation. It is fortunate that at this critical time the Board adopted a conservative policy. The method under which the Board operates is familiar to you. It has secured the approval and confidence of the agricultural people, and the first great step in the suppression of the infectious diseases of animals has been accomplished through education. Previous to 1896 more than $2500 per year had never been expended by the State for the suppression of diseases of the domestic animals. During the last two years about $90,000 has been expended without undue friction or unfavorable comment being excited from sources worth mentioning. In view of the success which has attended the efforts of the State Livestock Sanitary Board, we think it due this Association to express the most hearty commendation of their work. We extend to our sister States, which are endeavoring to have similar boards established, a word of encouragement with a hope that their efforts will soon be crowned with success.

The recent decision of United States District Judge Rodgers, at Kansas City, in which he held, in effect, that the present system of government meat-inspection was unconstitutional, has created quite a stir in legal and veterinary circles. It would appear, however, that the decision is one largely technical in character, which will not affect the practical operation of the law when applied to the inspection of meats intended for interstate traffic or export to foreign countries. We quote the opinion of Dr. Salmon, Chief of the Bureau of Animal Industry, who has direct supervision of this branch of the service, and who says: "The decision, I think, is rather technical, and Judge Rodgers is probably a close constructionist of the law. We rely on decisions of the United States Supreme Court for authority to show that the government has the right to inspect meats intended for interstate shipment. The intent of Congress when it enacted this legislation evidently was that the animal was a subject of interstate commerce from the time it was shipped from the State in which it was raised until reaching the destination for consumption. There is nothing for the department to do in the case as it now stands. If the meat is inspected for domestic consumption entirely within the limits of the State, then the United States authorities cannot insist on an inspection, but just so soon as it passes beyond the borders then inspection will be necessary, as this requisite is imposed by the requirements of the law. A large amount of our meats are now exported to Europe, and foreign countries will not accept them if not properly tagged and handled with the inspector's mark. Should the decision of Judge Rodgers be accepted literally by the proprietors of the packing-houses, and they should refuse to permit our inspectors to do their work as heretofore, we shall, when shipments reach the State boundaries, simply refuse to give a certificate of inspection. H. B. F.

Drs. W. H. Ridge, Chairman, H. B. Felton, W. S. Kooker, Otto G. Noack, Charles T. Goentner, Committee on Intelligence and Education.

The Committee on Sanitary Science and Police made a very commendable report, and the Chairman, Dr. J. Curtis Michener, made prophecies of the future's good work, much of which is sure to be realized:

*Report of Committee on Sanitary Science and Police.*

In this age of advancement, sanitary science is receiving its merited attention. We are now looking for the cause of disease, and no longer attribute it to an avenging Deity, except, when one of our own species succumbs, we usually acknowledge that it has pleased Almighty God to remove the loved one, regardless of the disease or accident that did it. There is still one very common (and in most cases senseless) cause given for attacks of sickness by the laity, and encouraged by the profession, that of taking cold; but grippe is quite fashionable, and both terms come handy when we do not know. It is encouraging that the most dangerous of the contagious and infectious diseases of both man and beast are now amenable to law or the regulations of health and livestock sanitary boards. Let us see to it that no retrograde step is taken, but press manfully onward until the profession becomes the appointed guardian, advising and protecting the people and annihilating disease germs. We are not dreaming or dealing with impracticable or visionary schemes, but are living witnesses of glorious victories being achieved. Bovine contagious pleuro-pneumonia is a thing of the past with us. Splenic-fever is being driven to narrow confines; only a few years ago it killed a thousand where it now destroys one. Glanders is growing far less prevalent as its victims fall into the hands of authority—only twenty-two cases reported within our State the past year. We used to meet twice that number in private practice. The percentage of cases responding to the tuberculin-test for our State Livestock Sanitary Board has declined from forty to thirteen, showing that progress is being made in fighting the disease. Tuberculosis is contagious; by that means it is propagated and kept among us. I discern the time when the insidious enemy will no longer be allowed to invade living bodies. Swine-plague, chicken-cholera, roup, and gapes are still allowed to kill their thousands. They can and will be stayed. Science will reveal the hidden enemies, and authority take hold and bring them to execution. It is time that we realized that sanitary science and police have a glorious destiny, and take hold with willing hands, and not be discouraged or dismayed by the opposition that ignorant conservatism is ever ready to give.

The general health of livestock in our State is good, judging from the meagre reports received. Dr. N. E. Reinhart, of Pottstown, gave an interesting report of an outbreak of spinal meningitis just too late for our fall meeting, and other cases have occurred; but, sorry to say, no new light has been shed upon this fatal disease. Dr. Francis Bridge reports much more azoturia, and many more recoveries, owing, probably, to the cases not being so acute. A decrease in tuberculosis and osteoporosis. Dr. J. B. Irons, of Erie, reports the Messrs. Eagley Brothers, of North Springfield, as having some trouble with their calves. One at the age of sixteen days showed an enlargement on side of lower jaw, remaining hard and enlarging, animal losing appetite; died when six weeks old. A piece of lung sent him looked like tuberculosis. One other showed similar symptoms, and was destroyed, and still another was reported to him. He tested the herd with tuberculin, but did not get the required reaction to condemn, and is unable to account for the similarity of the calves one after the other. We have been called in consultation in reference to some calves which have been developing a lung-trouble one after another the past two years, some dying and others making a very tedious recovery. The disease has been confined to those housed in one large box-stall, others on the farm escaping. We posted one and found the lungs hepatized in circumscribed patches, indicating the lodgment of a specific germ. A portion of diseased lung was placed in a sterilized jar and sent to the Veterinary Department of the University of Pennsylvania.

We have noted with pleasure the satisfactory working of our State Livestock Sanitary Board, and that its regulations are meeting with the approbation of our citizens as they become fully understood. May the good work go on supported by us all.

J. CURTIS MICHENER,
Chairman.

The local committee of arrangements, consisting of Drs. Kooker, Williams, Houldsworth, Marshall, Hart, and Gladfelter, were assiduous in their duties; and the noon-hour luncheon each day was a very enjoyable feature, much appreciated by all for its bounteousness and the opportunity it afforded for closer social relations of the members and their guests.

The final report of the Committee on Revision of the Constitution and By-laws was received and adopted as a whole, and 1000 copies ordered to be printed.

For three hours on the first day those present were favored by a number of operations by the members, with clinical lectures preceding, during, and subsequent to the operations. Dr. F. F. Hoffman, of Brookville, performed ovariotomy on a mare per vagina. Dr. Alex. Glass, of Philadelphia, ovariotomy on a bitch per linea alba of abdomen. Dr. S. J. J. Harger, of Philadelphia, performed median neurectomy and exhibited the section of limb when performed.

At 7.15 P.M. Dr. M. P. Ravenel, Bacteriologist of the State Livestock Sanitary Board, gave an unusually interesting and instructive illustrated lecture on bacteriology. The lantern-slides thrown upon a large canvas proved of the deepest interest and created the utmost eagerness among all present to know of the work and results of this new line of work by our State.

Never to be forgotten in memory or blotted from perceptive impression was the sumptuous dinner given by State Veterinarian Pearson in the University Restaurant Hall, where, around a horseshoe-shaped table, extending almost the entire length of the hall, beautifully and tastefully decorated, while the arched roof sent forth the draped colors of the State's University, gathered the largest number of veterinarians ever assembled around a banquet table at one time in our country. At the head sat the host, Dr. Leonard Pearson, faced by President Rayner, and in close touch were to be noted Drs. Ackerman, of Brooklyn, N. Y.; Lowe, of Paterson, N. J.; John Faust, of Poughkeepsie, N. Y., and around the table at divergent points the officers intertwined with the guests, numbering nearly one hundred and thirty. From oysters to wild fowl every course was enjoyed, and on the arrival of the cigars, the host, as President of the Keystone Veterinary Medical Association, called that body to order, as it was its regular monthly meeting, and Secretary Rhoads as promptly moved to adjourn, which was carried, when the host called upon Dr. W. Horace Hoskins, seated on President Rayner's left, to take charge as toast-master of the closing hours of the evening's feast. In a happy frame of mind at the honor of so unexceptionable an occasion, he could not forego the opportunity of fittingly referring to the successful work, broad interest, and fraternal spirit engendered by Pennsylvania's having so fortunately had added to her list of veterinarians our honored host, and with much feeling referred to the advances all along the line from the touch of one so peculiarly fitted for his vocation, and of the hour of pride at home and in every sister State when the great University of Pennsylvania selected him as the Dean of her Veterinary Department.

Calling upon Dr. E. B. Ackerman, of the Brooklyn Board of Health, to respond to the importance of the work of veterinarians associated with such bodies, brought forth an appreciative response of the pleasure of being present under such pleasant circumstances, the growing character of this work in cities and towns, and its value from a public health standpoint.

Why one good strong association does not exist in New Jersey was fully explained by Dr. Wm. Herbert Lowe, of Paterson, N. J., and why every phase of sanitary and public health work is done in a divided manner, was forcibly referred to.

From that sage of the profession, whose advancing years makes more appreciative his love for his calling, came sound words of advice that were applauded to the echo and referred to by younger speakers, upon the response of Dr. John Faust, of Poughkeepsie, N. Y.

In responding for the medical profession our sister vocation, Dr. Detweiler, of Williamsport, Pa., told wittily and fittingly how veterinary sanitary measures governing the production and care of milk were controlling the diseases of children, and if they kept on many of his calling would be out of service in the summer season completely.

Dr. John W. Adams, of the Veterinary Department, took as his text the wholesome advice given by Dr. Faust, and strongly urged upon his every hearer to be ever a student, always a searcher for truths, and to complete in the succeeding years the studies that were only "how to learn" commenced in college days.

Dr. M. E. Conard, of West Grove, Pa., told of the new sphere of work for veterinarians among large milk handlers, and in fitting words paid a splendid tribute to the work of Pennsylvania's Livestock Sanitary Board and to the conservative direction given it by our honored host.

Official place in association work was the theme of an inspiriting address by Secretary Rhodes, and the duties of members to officers was thoughtfully alluded to in strong words.

Canine practice in large cities proved an at-home subject to Dr. Alex. Glass, of Philadelphia, and its growth and importance were well presented. The opportunity of speaking to such a body of his colleagues seemed to fill the speaker with many happy thoughts of college days as a student, and later as an instructor, and traits of character, special abilities noted in students' careers, were happily treated upon.

Secretary Harger, of the Pennsylvania State Veterinary Examining Board, spoke of the value of constant study and application and how one was to better equip himself for specialized work.

Followed by Dr. J. C. McNeil, of the Department of Public Safety of Pittsburg, who thought this was a good time and opportunity to urge the consideration of the "Smoky City's" claims for the next annual meeting in September, and to promise every protective care to all who would come and bask in their hospitality.

Representing a family of whom three generations yet remain in practice, Dr. J. Curtis Michener, of Colmar, after a brief reference to State work, rendered an amusing recitation, which had an added interest to the younger single element around the board.

After a number of other brief responses by State representatives, the closing remarks were called for from our esteemed host, who, in speaking of the pleasure of seeing so many faces around the board, was somewhat embarrassed as to how best he could tell of the personal gratification of such a response to his invitation, and closed with a good-night wish for all.

The seond day's session was started promptly at 10 A.M., though many were a little late. The attractions of the Veterinary Department and the Experimental Buildings and Laboratory of the State Livestock Sanitary Board proved very great attractions to all the visitors from out of the city. After a brief period of routine work, came the reading of the papers, and President Rayner called upon Dr. M. E. Conard, whose paper on "What Our Dairy Cattle Inherit?"[1] brought out some very salient points and wiped away many popular prejudices and unsound theories that prevail.

As several other papers on the programme were on kindred subjects, it was decided by a motion to have these read and then take up their discussion severally.

Dr. H. B. Felton, in considering the value of "Pasteurization *versus* Purity,"[2] presented an excellent paper bearing upon the importance and care of milk preparations, and brought out very clearly the barriers of defense from disease.

"Feeding of Animals,"[3] by Dr. J. Curtis Michener, proved a very practical and pointed paper. The importance of veterinarians fitting themselves to be capable of giving advice and assistance was well told and appreciated by all who heard this paper read.

"What a Veterinarian Ought to Know about Commercial Milk,"[4] proved to be well understood by the author, Dr. C. J. Marshall, and many valuable formulas of modified milk for young animals were presented, of much interest and importance.

Dr. M. P. Ravenel, Bacteriologist of the State Livestock Sanitary Board, followed with one of the most important papers of recent date: "Milk Supply from a Bacteriological Standpoint."[5] Its careful preparation, the thermal death-points of the many germ forms of life injurious to health, its statistics, showing the milk consumption in several large cities, and many other points of great importance provoked an extremely important and instructive discussion, which lasted for several hours, and was participated in by Mr. G. H. Gordon, of the Walker-Gordon Laboratory; Mr. George Abbott, the largest and foremost individual milk-dealer of Philadelphia; Dr. J. Cheston Morris, proprietor of the Devon Milk Dairy, and who twenty-five years ago adopted the sealed-jar system of delivering milk, filling the same at the point of production; Mr. S. M. Brosius, of Chester County, connected with one of the large milk exchanges of Philadelphia; Dr. E. B. Ackerman, of the Brooklyn Board of Health, and by the authors of the several papers. The value of the various constituents of milk from a dietetic point of view came in for a round of discussion, and the claims for a milk rich in fats, one rich in the other solid constituents one of moderate richness as to fats, but prepared under the most rigid care as to cleanliness, were fully dilated upon, and from every speaker came words of praise and support to the veterinary profession for their activity and interest

1 See p. 237 of this issue of the JOURNAL. 2 Ibid., 245. 3 Ibid., 253.

4 Ibid., 232. 5 Ibid., 215.

in this all-important subject, so that every one present felt it was doubly good to have been there.

Secretary Rhoads could not refrain from reading a telegram from the newly elected President, Dr. George B. Jobson, who in the most earnest manner assured the members of the very high appreciation of the honor they had conferred upon him.

Secretary Rhoads, in the absence of Drs. Phillips, Phipps, Bland, and Keely, read their papers, entitled "Tetanus,"[1] "Tetanus as I Have Found it in Chester County,"[3] "The Value of Blistering with Mercurial Preparations in Preference to Actual Cautery,"[2] and "Eversion of the Uterus in Cows."[4]

These were followed by the presentation of a paper by Dr. S. J. J. Harger on "Lesions of Spavin."[5] This paper was a very practical one, and was further elucidated by presenting several dried specimens and a number of hock-joints.

Dr. J. F. Butterfield reported "A Case of Distressed Breathing in a Cow, and its Relief by Mechanical Adjustment of a Tracheal Tube."[6] The result obtained was very satisfactory and its application unique.

Other cases were reported by Dr. F. F. Hoffman and others, after which the Committee on Resolutions presented the following:

*Report of Committee on Resolutions.*

WHEREAS, The Governor of Pennsylvania, Honorable Daniel H. Hastings, has in many ways shown his appreciation of the importance of questions relating to the agricultural and health interests of the people; and

WHEREAS, This interest has been expressed by many acts of great value to the Commonwealth, and especially in connection with his organization of the State Veterinary Medical Examining Board, which has had his unvarying support; in the appointment of Dr. Benjamin Lee, a trained, efficient, and experienced sanitarian, as Health Officer at the Port of Philadelphia, and in his constant interest and assistance in the work of the State Livestock Sanitary Board; be it

*Resolved*, That we hereby tender Governor Hastings this testimonial of our deep appreciation and sincere respect; and be it further

*Resolved*, That a copy of these resolutions be presented to the Governor; also, that the resolutions be spread upon the minutes of the Pennsylvania State Veterinary Medical Assocation, and published in the journals of the profession.

WHEREAS, There has recently been some opposition in Philadelphia to the sale of milk in bottles, and this opposition is based on the alleged grounds that this method tends to favor the spread of certain infectious diseases among consumers of milk, and that it interferes with the proper inspection of milk by the officers of the Health Board of Philadelphia; and

WHEREAS, Although this method of distributing milk has been in use many years (in one case twenty years under the most careful observation), and there is no evidence to show that it has resulted in the transmission of disease as feared, and it must be perfectly evident to all who have any practical knowledge of the subject that it is much easier to protect milk from contamination and to keep it cold, thus checking bacterial growth, when it is in sealed bottles than when it is in large cans that are frequently opened; and

WHEREAS, We do not recognize the method of milk-inspection that is at present practised in Philadelpia, namely, a chemical determination of the

[1] See p. 266 of this issue of the JOURNAL. [2] Ibid., 256. [3] Ibid., 265.

fats and total solids in milk, as the one that is of the greatest importance to the public; and

WHEREAS, The present agitation, originating largely through a trade dispute among milk-dealers, has tended to unduly lessen confidence in the milk-supply of Philadelphia, with the possibility that this may react to their own disadvantage and the disadvantage of the producer; therefore, be it

*Resolved*, That the Pennsylvania State Veterinary Medical Association approves of all changes and advances in methods of milk-distribution that tend to bring this important article of food to the consumer with additional guarantees of purity, wholesomeness, and cleanliness; that we regard the health of the cows that produce it as of first importance; and a proper system of milk-supply includes not only this, but also clean, wholesome food and surroundings for the cows, careful milking, clean attendants, sterile vessels, provision for thorough cooling and rapid transportation to the consumer; be it further

*Resolved*, That we recognize no intrinsic objection to the system of selling milk in bottles; but, on the contrary, regard this system when carried out in a proper manner, including the thorough cleansing and sterilization of the bottles after use, as a distinct advance in methods of milk-distribution; be it further

*Resolved*, That a system of milk-inspection based upon chemical examination alone is incomplete and insufficient, because it omits the points that are of most vital interest in the protection of the public health, and we do not believe in a system of inspection that interferes with the proper development of methods of milk-supply.

WHEREAS, This Association, in convention assembled, has heard from every section of our State the warmest expressions of approval of the work of the State Livestock Sanitary Board; and

WHEREAS, This work is growing in importance and is now recognized as of the utmost value to the entire population of our Commonwealth, this recognition being largely due to the conservative methods adopted by those charged with the duties of executing the laws relating to diseases of animals and the regulations of the Board; and

WHEREAS, The results of this work in the suppression and control of communicable diseases have been so marked, and the expectations of the most sanguine have been so fully met, that the most skeptical of those who questioned the wisdom of the establishment of this work in our Commonwealth have been convinced of its value; and

WHEREAS, These results have come largely from the earnest personal support and assistance given the State Livestock Sanitary Board by the Governor, the Secretary of Agriculture and the Dairy and Food Commissioner and by our colleague, Dr. Leonard Pearson, the efforts of all of whom excite our warmest approval and commendation; be it

*Resolved*, That we express to this Board our most hearty indorsement of its thorough yet conservative methods, and of our gratification in the results already obtained, and our confidence that greater results will follow the enforcement of the broader and better plans that are in prospect.

WHEREAS, A bill is now pending before Congress that will, if enacted into law, seriously interfere with and in many cases utterly prevent research work in bacteriology, pathology, and physiology in the District of Columbia; and

WHEREAS, It is of the highest importance that work in these lines under the direction of the Federal Government shall not be interfered with for the reason that it not only promises valuable developments in the future, but that it has already yielded results that have been the means of saving untold millions of dollars, and has greatly advanced public sanitation and preventive medicine. We recognize the bacteriological and pathological work of the Bureau of Animal Industry is among the most important that has been done anywhere in the world; therefore, be it

*Resolved*, That the Pennsylvania State Veterinary Medical Association is strongly of the opinion that Senate Bill No. 1156 is calculated to retard and injure work directed to the prevention of suffering and disease, and we request the members of Congress from Pennsylvania to vote and use their influence against it; and be it further

*Resolved*, That the Corresponding Secretary is hereby instructed to send a copy of these resolutions to each member of Congress from Pennsylvania.

WHEREAS, This our sixteenth annual meeting has proved to be the most successful and largest in our history; and

WHEREAS, Nothing has contributed more to the pleasure and comfort of all who were able to be present than the very attractive and commodious quarters and facilities afforded us by the University of Pennsylvania Veterinary Department; be it

*Resolved*, That we gratefully acknowledge our very great indebtedness to this Department for these very great privileges.

WHEREAS, On this most enjoyable occasion and annual reunion we have been honored and feasted by our esteemed colleague, Dr. Leonard Pearson; and

WHEREAS, This special pleasure long to be remembered with many happy and pleasant memories; therefore, be it

*Resolved*, That we acknowledge our most profound thanks to our colleague and co-laborer for this token of his personal esteem, and tender to him our sincere good wishes for his future good health, success, and prosperity.

WHEREAS, Deeply mindful of the great responsibilities of official place in such organizations as this, and remembering the labor incidental thereto; be it

*Resolved*, That we tender our sincere thanks and appreciation to our retiring officers, and wish for their successors an equal measure of prosperity in Association interests and advancement.

WHEREAS, The growing scope of work done by our Association is yearly measured by a more and more attractive programme afforded by our members; and

WHEREAS, We have been specially favored this year by a most excellent and varied programme; be it

*Resolved*, That we hereby tender our most appreciative expression of thanks to all of our members who have favored us with interesting reports and papers, and especially to our esteemed co-laborer in comparative science, Dr. M. P. Ravenel, for his interesting, instructive and entertaining lecture.

WHEREAS, The local committee of arrangements representing the veterinarians of Philadelphia, have been zealous in their efforts to provide for the comfort and welfare of all in attendance at our convention; and

WHEREAS, Their efforts have been crowned with success and contributed so much to the pleasure of this occasion; therefore, be it

*Resolved*, That we in the enjoyment of the same, desire to express our very great appreciation of their generosity and kindness.

WHEREAS, This Association has on past occasions found itself very greatly obligated for the kindness and courtesy of the press of Philadelphia; and

WHEREAS, On this our sixteenth annual meeting it has been so well and thoroughly reported in our daily press; be it

*Resolved*, That we extend to the press of Philadelphia our very great appreciation of their uniform courtesy; their interesting reports of our sessions, and the excellent work they are doing in educating public opinion of the need and importance of better food inspection.

These were adopted by a unanimous vote.

The place of meeting for the semi-annual gathering brought out the friends of Pittsburg, Gettysburg, and several other towns, but the earnest solicitation of Dr. McNeil, of the "Smoky City," and his determination to have a grand meeting in that city, won the honor, and westward will be the word in September.

The seating of those of the newly elected officers who were present followed, with many appropriate and fitting remarks from the outgoing and incoming, after which, the hour being late, the meeting adjourned, thus closing Pennsylvania's greatest, most profitable, and pleasant gathering, all returning home feeling a renewed interest in the profession's advancement and its needed work.

